# From ex vivo to in vivo chimeric antigen T cells manufacturing: new horizons for CAR T-cell based therapy

**DOI:** 10.1186/s12967-024-06052-3

**Published:** 2025-01-04

**Authors:** E. Pinto, L. Lione, M. Compagnone, M. Paccagnella, E. Salvatori, M. Greco, V. Frezza, E. Marra, L. Aurisicchio, G. Roscilli, A. Conforti

**Affiliations:** 1 Evvivax Biotech, Via Castel Romano 100, 00128 Rome, Italy; 2 Department of Clinical Sciences and Translational Medicine, University of Rome “Tor Vergata”, Via Montpellier 1, 00133 Rome, Italy; 3 Takis Biotech, Via Castel Romano 100, 00128 Rome, Italy

**Keywords:** CAR T cell, Manufacturing, T cell engineering

## Abstract

In the past decades, Chimeric Antigen Receptor (CAR)-T cell therapy has achieved remarkable success, leading to the approval of six therapeutic products for haematological malignancies. Recently, the therapeutic potential of this therapy has also been demonstrated in non-tumoral diseases. Currently, the manufacturing process to produce clinical-grade CAR-T cells is complex, time-consuming, and highly expensive. It involves multiple steps, including the collection of T cells from patients or healthy donors, in vitro engineering and expansion, and finally reinfusion into patients. Therefore, despite the impressive clinical outcomes, ex vivo manufacturing process makes CAR-T cells out of reach for many cancer patients. Direct in vivo engineering of T cells could be a more rapid solution able to circumvent both the complexity and the costs associated with ex vivo manufactured CAR-T cells. This novel approach allows to completely eliminate ex vivo cell manipulation and expansion while producing therapeutic cell populations directly in vivo. To date, several studies have demonstrated the feasibility of in vivo T cell reprogramming, by employing injectable viral- or nanocarrier-based delivery platforms in tumour animal models. Additionally, in vivo production of CAR-T cells might reduce the incidence, or at least the severity, of systemic toxicities frequently occurring with ex vivo produced CAR-T cells, such as cytokine release syndrome and immune effector cell-associated neurotoxicity syndrome. In this review, we highlight the challenges associated with the current ex vivo manufacturing protocols and review the latest progresses in the emerging field of in vivo CAR-T therapy, by comparing the various platforms so far investigated. Moreover, we offer an overview of the advantages deriving from in vivo reprogramming of other immune cell types, such as Natural Killer and macrophages, with CAR constructs.

## Introduction

Since the first idea of genetically engineering T cells with a novel chimeric receptor, demonstrating antigen recognition by T cells in an MHC-independent context and overcoming the limitations of TCR-engineered T cells, excellent results have been achieved. This is particularly evident in haematological malignancies such as leukaemia and lymphoma, leading to the FDA approval of CAR-T cell-based products.

However, along with the outstandings therapeutic achievements of CAR-T cell treatment, this therapy also faces multifaceted challenges, such as the complex and expansive manufacturing process and, in addition, several systemic toxicities.

Recently, the generation of other immune cells such as chimeric antigen receptor-modified macrophages (CAR-M) and natural killer cells (CAR-NK) has been explored as promising alternatives. These approaches are being considered due to their potential advantages, such as better infiltration into solid tumours, lower risk of graft-versus-host disease (GvHD) and reduced systemic toxicities. The in vivo engineering of these cells presents new opportunities to further simplify the manufacturing process and enhance therapeutic outcomes.

In this regard, the purpose of this review is to describe the challenges associated with current ex vivo CAR-T cell-based therapeutic approaches, while providing an overview of the recent advances achieved through the strategy of in vivo engineering of T cells, macrophages, and NK cells for CAR-based therapies.

## Car structure

**Fig. 1:**
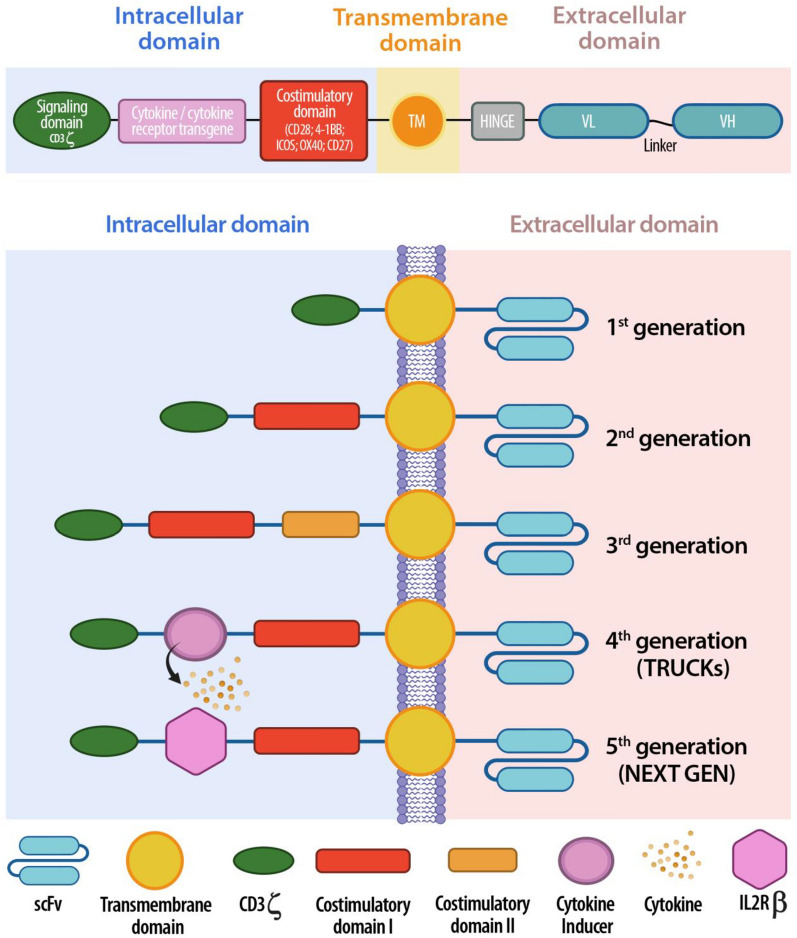
**A** Schematic representation of CAR-T cell structure: intracellular, transmembrane, extracellular domain. **B** Evolution of the five generation of CARs: from the first generation, containing only one activation domain, to the last next generation CARs, aiming to improve their safety and efficacy

CAR constructs have a typical modular design consisting of three major domains: ectodomain, transmembrane domain and endodomain, each of them composed of several functional structures (Fig. 1A).

The ectodomain is the extracellular structure responsible of the recognition of antigens expressed by tumour cells in an MHC-independent manner, ensures strong activation of CAR-T cells and consists of antigen-binding domain and hinge domain. Single chain variable fragment (scFV) are the most commonly used antigen-binding domains in CAR design. However, their use can have drawbacks, such as immunogenicity and a tendency to self-aggregate, which can lead to early exhaustion of CAR-T cells [1]. As an alternative, antigen-binding domain can be composed of nanobodies, also called VHH domain, able to access solid tumour-associated epitopes, normally hard to be reached by scFVs [2]. The Hinge domain (HD), also known as “spacer”, connects the antigen-binding domain beyond the plasma membrane, being anchored to the transmembrane domain. HD provides stability and flexibility to the CAR, and its choice is a crucial step when designing CARs. In this regard, it has been demonstrated that the use of Ig-based hinge domains might trigger innate immune responses, because of the ability of CH2 region of IgG molecules to bind to Fcγ receptors expressed by innate immune cells [3]. However, mutation or complete deletion of CH2 in Ig-based hinge can reduce immune reactions and improve CAR-T cells persistence and antitumor efficacy [4]. HD length also must be carefully designed, basing on the epitope distance from the cell membrane to be achieved: longer hinges are able to provide more flexibility and allow access to membrane-proximal epitopes or complex glycosylated antigens, whereas shorter hinges can be employed to target membrane-distal epitopes [5, 6].

The transmembrane domain (TMD) of CARs connects the ectodomain to the intracellular signalling domain. Although the primary function of the TMD is structural, the expression level and stability of CARs on T cells can be affected by the type of TMD used [7]. Moreover, TDM can also be relevant for CAR-T cell function, since it is involved in CAR dimerization, upon antigen engagement by the antigen-binding domain, and mediates the transmission of the activation signal by interacting with endogenous signalling molecules [8]. Therefore, the choice of the type of TDM allows to modulate CAR functions.

The endodomain is the cytoplasmatic structure responsible for the intracellular signalling by transmitting the external antigen recognition signals to the inside of the cell to activate the T cell and trigger an immune response [9]. It generally contains a T cell activation domain and one or multiple co-stimulatory domains. The most commonly used activation domain, employed in all FDA-approved CARs, is the CD3ζ chain, a component naturally found in the TCR complex [10]. Immunoreceptor Tyrosine Activation Motifs (ITAMs) present on cytoplasmic domains of the CD3ζ chain are the phosphorylation sites recruiting ZAP70, a protein kinase involved in signalling cascades [11]. Although CAR-T cells form non-classical immune synapses, they have also been demonstrated to activate the ZAP70 kinase upon antigen recognition [12]. Several types of costimulatory molecules and other functional domains in addition to the CD3ζ chain have been widely investigated over the years, leading to the development of different generations of CARs that are thoroughly described in the following paragraph.

## CAR design evolution: from the first to the “next generation” CARs

By developing the so called “T-bodies” in 1987, Kuwana et al. were the first to elucidate the key concept underlying CAR T-cell therapy, by combining portions from monoclonal antibodies with the TCR [13]. The aim was to generate T cells able to recognize tumour antigens in an MHC-independent manner, given to the presence of antigen-binding site from monoclonal antibodies, and to eradicate tumour cells upon activation of the TCR signalling pathway. The first concept of CAR-T cell was then illustrated by Gross and Eshhar in 1989, who fused the heavy and light chain of a monoclonal antibody to the TCR [14, 15]. Although their basic conformation has remained the same since their inception in the late 1980s, CARs have gone through five generations in the last decades (Fig. 1B), each one characterized by modifications to the structure, composition, and function of the intracellular domain, with the aim to increase activation, persistence, proliferation, safety and efficacy [16].

First-generation CARs were composed by a scFv and an intracellular signalling domain containing the Fc receptor gamma chain (FcγR) or the CD3ζ chain alone. Early studies comparing these signalling motifs showed superior cytotoxic activity in CD3ζ -based CAR-T cells compared to FcγR-based CAR-T cells, probably due to the presence of multiple ITAMs in the CD3ζ chain [17]. However, first-generation CARs demonstrated to have poor persistence and low antitumor efficacy in several clinical trials, although cytotoxicity against target cells was observed in vitro and in preclinical studies [18]. Therefore, given the importance of co-stimulation for durable CAR-T cell therapy, second-generation CARs containing a costimulatory molecule, placed in-*cis* with the CD3ζ into the intracellular signalling domain, were developed. The most characterized and commonly used costimultatory molecules belong to the CD28 receptor family, such as CD28 and inducible T cell co-stimulator (ICOS), or the tumor necrosis factor receptor family, such as 4-1BB (CD137), OX40 (CD134). Upon interaction with its cognate ligands CD80 (B7-1) and CD86 (B7-2) expressed on APCs, CD28 signalling enhances IL-2 production, survival, proliferation and metabolic activity of naïve T cells, so improving CAR-T-cell survival, function and antitumor response [19, 20]. 4-1BB is an inducible T cell surface receptor belonging to the tumour necrosis factor receptor superfamily, whose expression normally lacks on naive T cells, since it is only induced upon T cell activation [21]. Similar to 4-1BB, OX40 is known as a late costimulatory molecule that, upon the binding with its ligand OX40L expressed on activated APCs, promotes T cell persistence and the generation of memory T cells [22]. ICOS is expressed on activated T cells and its binding to ICOSL is crucial to direct immunity towards humoral or inflammatory responses and for the development and maintenance of human T helper 17 (Th17) cells [23]. The addition of costimulatory molecules in second-generation CARs improved T cells proliferation, activation, cytotoxicity, and in vivo persistence of CAR-T cells, but different downstream pathways can be induced based on the type of costimulatory molecule included into the CAR design. Indeed, CARs bearing CD28 costimulatory domain could lead to greater T cell expansion, survival and quicker antitumor response, compared to CARs bearing 4-1BB [24, 25]. However, quicker activation might induce tonic signalling and T cell exhaustion and, therefore, lack of durable antitumor response. On the contrary, despite poorer in vitro efficacy and the slower in vivo expansion compared to CD28, 4-1BB-based CARs induced superior long-term persistence [26, 27] and a central memory phenotype, with a lower degree of phosphorylation and weaker tonic signaling, relying on fatty acid metabolism. Conversely, costimulation mediated by CD28 preferentially promotes effector memory phenotype development and aerobic glycolysis, a type of metabolism that is connected to rapid activation and more pronounced phosphorylation [28]. Similarly, CARs containing ICOS as costimulatory domain were shown to promote better persistence of CAR-T cells in vivo when compared to CD28-bearing CAR-T cells [29].

Even though the introduction of a single co-stimulatory molecule in second-generation CARs allowed to obtain significant improvements compared to those observed with first-generation CARs, the need to further improve clinical responses prompted the development of third-generation CARs. The aim was to optimize the different pathways induced by various costimulatory molecules by combining them into a single CAR. Therefore, third-generation CARs were obtained by including two different co-stimulatory domains, most commonly CD28 and 4-1BB, in addition to the CD3ζ chain, resulting in a “dual signalling”, in which the quick and powerful cytotoxicity efficacy belonging to CD28 molecule synergized with the 4-1BB ability to ensure durable CAR persistence and proliferation and more effective antitumor responses [30, 31]. Several preclinical studies demonstrated superior antitumor response and improved in vivo persistence of third-generation CAR-T cells, compared to second-generation ones, to treat different types of cancer [29, 32, 33]. Superior expansion and longer persistence were also observed in early clinical trials in patients with leukemia and non-Hodgkin lymphoma (NHL) [34]. However, Ramello et al. found that, compared to third-generation CARs, second-generation CARs can engage additional species of CD3ζ in a supramolecular signalling complex that may induce a more intense signalling and contribute to their superior antitumor capacity [35].

A fourth-generation of CARs, also named as “T cells redirected for universal cytokine killing” (TRUCKs), was developed to introduce additional transgenes for inducible cytokine release. The intracellular signalling domain of this generation is based on second-generation CARs but owns a nuclear factor of the activated T-cell (NFAT)-responsive cassette containing transgenic immunomodulators (such as IL-2, IL-12, IL-15, IL-18) and a second co-stimulatory domain. Upon the binding of TRUCKs to the tumoral antigen, activation of NFAT results in the productions of cytokines responsible for the recruitment of innate immune cells that, in turn, cooperate and enhance CAR-mediated antitumor response, specifically confined to the tumour microenvironment [36].

The most recent fifth-generation, also known as” next generation”, CARs are currently in active development and are designed to have multiple functions with the aim to obtain safer and more effective antitumor response. These CARs are based on second-generation CARs but incorporate an additional truncated cytoplasmatic domain derived from the beta chain of the IL-2 receptor (IL-2 Rβ), located between the CD28 co-stimulatory domain and the CD3ζ chain, and a binding motif for transcription factors like STAT-3/5. Following antigen binding, the simultaneously activation of the CD28 co-stimulatory domain, CD3ζ chain and JAK-STAT 3/5 pathway promotes CAR-T cell function, proliferation, and persistence [37].

Further improvements in the design of next-generation CARs aim to suppress immune checkpoint inhibitor (ICI) pathways, typically mediated by the binding of PD-1 or CTLA-4 to PD-L1 and CD80/CD86, respectively, in order to reduce CAR-T cells exhaustion and enhance antitumor activity [31]. Beyond this combinational approach, a novel strategy to block the ICI-mediated pathways by engineering CAR-T cells has been recently investigated and improved efficacy in terms of diminished exhaustion and increased antitumor response has been demonstrated. Interestingly, Liu et al. conducted a phase IB study to evaluate the antitumor efficacy of a CD19-specific CAR-T expressing PD-1/CD28 chimeric switch-receptor [38]. This novel receptor contains the extracellular domain of PD-1 fused to the transmembrane and cytoplasmic domain of the costimulatory molecule CD28 and therefore, upon engagement of PD-L1 expressed on tumour cells, triggers an activating signal (via the CD28 cytoplasmic domain) instead of the typical inhibitory signal mediated by the PD-1/PD-L1 pathway. When treated with this novel CAR-T product, termed as CD19-PD-1/CD28-CART, patients showed efficient and durable clinical response.

In addition, ICI receptors can be incorporated into CAR design to generate inhibitory CARs (iCARs) with the aim to circumvent on-target/off-tumour toxicity. iCARs recognize antigens that are expressed on normal tissues, but absent on tumour tissue, and are expressed along with CARs that specifically target antigen expressed on tumour cells or normal tissues. Upon the interaction with healthy tissues expressing the antigen recognized by the iCAR, the inhibitory signalling cascade triggered by inhibitory coreceptor coupled to the signalling domain of iCAR, such as PD-1 or CTLA-4, will prevent the activation of the CAR T-cell, leading to minimization of on-target/off-tumour toxicities [39].

Of note, despite the success achieved in treating haematological malignancies, no CAR T-based therapy has yet been approved for solid tumours [40], mainly due to the hostile tumour microenvironment (TME) and the lack of tumour specific antigens capable to minimize on-target/off-tumor toxicities [41]. As tumour stroma is abundantly composed of heparan sulfate proteoglycan (HSPG) and hyaluronic acid, CARs engineered with Heparinase [42] and Hyaluronidase [43] enzymes have been demonstrated to disrupt the TME by degrading hyaluronic acid and HSPG, thus improving CAR-T cells infiltration. Also, CARs engineered to express appropriate chemokines receptors binding to chemokines secreted by tumor cells, such as CXCR1 [44], CXCR2 [45] and CCR4 [46], have been demonstrated to improve CAR-T cell homing and trafficking toward multiple solid tumors. In order to reverse the immunosuppressive TME of solid tumors, armored CAR-T cells, secreting pro-inflammatory cytokines, such as IL-12 [47], IL-15 [48], IL-18 [49] and IL-7 [50] have been generated to improve antitumor activity and to directly target TME. Also, considering the high heterogeneity of antigens expressed by tumor cells, current CAR-T cell therapy for solid tumors usually target tumor-associated antigens (TAAs) that often undergo the phenomenon of antigen escape and evade CAR-T cell detection. Dual-targeting CAR-T cell therapy, based on CAR-T cells able to recognize two or more TAAs, represents a promising approach to overcome tumour heterogeneity and antigen escape characterizing solid tumours. There are several strategies to obtain multi-antigen specific CAR-T cells. The first approach, named “pooled CAR-T cells”, consists in administering two or more different CAR-T cells together, each targeting a single antigen. Administration of pooled CAR-T cells targeting EphA2 and FAPα showed superior antitumor response compared to either CAR in a murine xenograft model of lung cancer [51]. Similar outcomes were observed in a Non-Small Cell Lung Cancer (NSCLC) model treated with pooled prostate stem cell antigen (PSCA)- and MUC1-targeting CAR-T cells [52]. Bispecific CAR-T cells (biCAR-T) represent an alternative approach, in which a single T cell is engineered with two CARs directed against two tumour antigens. biCAR-T cells co-expressing HER2 and IL13Rα2 CAR molecules induced enhanced antitumor response compared with unispecific CARs alone or pooled HER2-CAR-T and IL13Rα2-CAR-T cells in a glioblastoma model [53]. Alternatively, T cells can be engineered with one CAR able to recognize two different antigens (bivalent CAR), due to the presence of two scFVs. Bivalent CARs can be classified in two different types: in “tandem” bivalent CARs, the variable light-heanvy chain complex (VL-VH) of one scFv is directly linked to the VL-VH of the other scFv, while in “loop” bivalent CARs the VL-VH of one scFv is separated by the VL-VH of the other scFv [54].

Dual CAR-T cells are also referred as CAR-T using “OR” logic gate, meaning that they that can be activated by either one or more of the targeted tumour antigens, whereas “AND-gate” CARs can be activated only when two different tumour antigens are targeted, with the aim to minimize on target-off tumor toxicity and improve safety in clinical translation.

## Challenges and limitations of ex vivo CAR-T cell-based therapy

### Manufacturing process

The manufacturing and administration processes of CAR-T cells are considered the primary contributors to the high costs of CAR-T cell-based therapy [55]. CAR-T cell manufacturing is highly expensive and time-consuming and, since the first approved CAR-T cell product by the US Food and Drug Administration in 2017 (namely, Tisagenlecleucel) for the treatment of B cell Acute Lymphoblastic Leukemia (B-ALL) [56], concerns raised about the broadly accessibility of CAR-T cell therapy for health care systems and for patients with rapidly progressive or aggressive cancers. Preparation of CAR-T cells, according to the classical ex vivo protocol, requires about 3–4 weeks and is accompanied by expenses typically over $400,000 USD and sometimes over $1 million USD per patient [57, 58]. It is difficult, therefore, to consider this therapeutic method as a standardly applicable clinical treatment for cancer diseases.

**Fig. 2:**
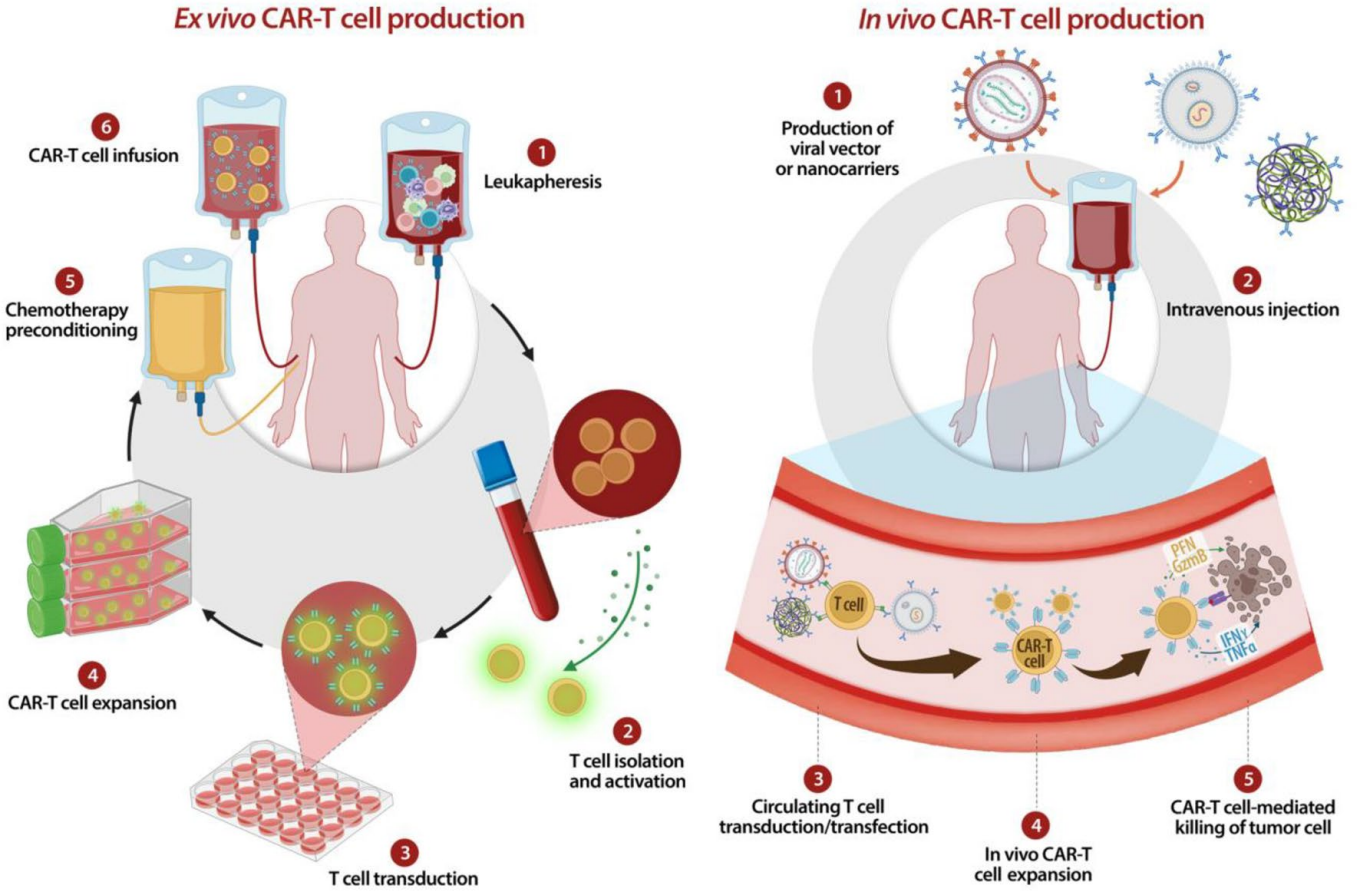
Ex vivo* vs *In vivo CAR-T cell production. In this illustration, ex vivo CARs generation passages are compared with in vivo CARs production by intravenous injection of viral vectors or nanocarriers

All current clinically approved CAR-T cells are autologous products, obtained by ex vivo engineering of patient-derived T cells (Fig. 2). The autologous CAR-T cell manufacturing process comprises the following steps: (a) leukocytes collection from the patient peripheral blood via leukapheresis; (b) activation and transduction of T cells using viral or non-viral vectors; (c) expansion of transduced T cells in cytokine-supplemented culture medium; (d) concentration of T cells and (e) CAR-T cells reinfusion into patient. Finally, for all approved CAR-T cell therapy, defined product characteristics, such as sterility, cell viability, purity, potency, identity and stability, must be downstream evaluated [59, 60].

Like all personalized cellular therapies, autologous CAR-T cells face multiple challenges, such as logistic expenses for leukapheresis collection, manufacturing and rapid shipment given to short shelf life of CAR-T products [55, 61].

Because of the difficulties associated with the patient-specific nature of the autologous CAR-T cells and complex manufacturing process, allogeneic CAR-T cells have been proposed as an alternative approach to reduce manufacturing costs. According to the allogeneic approach, healthy donors-derived T cells, usually isolated from PBMCs, are engineered using CAR-encoding viral or non-viral vectors. Allogeneic T lymphocytes manufacturing offers fully functional CAR-T cells in high amounts allowing multiple generations of “off-the-shelf” CAR T cells products [62, 63]. By overcoming many of the limitations associated with autologous CAR-T cells manufacturing, the development of this ‘off-the-shelf’ product aims to enhance scalability and direct access to CAR-T therapies and, therefore, to provide a readily available therapeutic solution [64].

Although allogeneic CAR-T therapy could reduce the manufacturing time and costs associated with the autologous one, downstream side effects must be considered: TCR present on the surface of allogeneic CAR-T cells could recognize and attack patient’s healthy tissues, thus triggering a potential fatal toxicity called “graft-versus-host” (GvH) [65]; conversely, in the scenario of the “host-versus-graft” (HvG) reaction, the patient’s immune system might rejects the foreign allogeneic CAR T cells and limit their in vivo persistence, thereby making the treatment inert [66].

### CAR-T cell-associated toxicities

Along with the remarkable results in treating cancer diseases, CAR-T cell therapy is associated with severe toxicities, such as cytokine release syndrome (CRS) and immune effector cell-associated neurotoxicity syndrome (ICANS).

CRS is a systemic inflammatory response characterized by “cytokine storm” due to massive T cell stimulation triggered by different factors, such as infectious diseases or certain drugs [67]. The exact pathophysiology of CRS is not yet completely understood, but CAR-T cell administration dose can affect the severity and the insurance of CRS and ICANS, as observed in patients of a clinical trial treated with different doses of infused CAR-T cells [68]. Regarding lymphodepletion regimen prior to CAR-T cells infusion, it was observed a higher incidence of CRS in patients preconditioned with cyclophosphamide and fludarabine [69]. Correlation between CRS insurance and lymphodepletion preconditioning is likely due to the fact that the latter influences the cytokine milieu by eliminating cells that act as sinks for those cytokines that support CAR-T cell function and proliferation; in turn, the increasing availability of these pro-survival cytokines leads to excessive expansion and activation of CAR-T cells [70–72].

ICANS is defined as neurotoxicity associated with immune effector cell therapies [73]. In the case of CAR-T cell therapy, ICANS typically occurs after the development of CRS and is correlated with the severity of CRS [74, 75]. Pathophysiology of ICANS relies on abnormal systemic inflammatory response triggered after activation of infused CAR-T cells and is characterized by increased levels of proinflammatory cytokines and marked endothelial activation. To date, there remain no approved therapies for the prevention of the above toxicities. Therefore, a comprehensive understanding of CAR-T therapy-associated toxicities, including their etiology and mechanisms, so as the optimization of CAR engineering, are imperative to effectively manage, treat, or mitigate toxicities occurrence in upcoming CAR-T therapies [76, 77].

## In vivo CAR-T cell generation: an attractive alternative

Because of the aforementioned drawbacks associated with current CAR T cell-based therapy, the need to investigate novel strategies to overcome the complexities of the ex vivo CAR-T manufacturing process and the associated systemic toxicities is increasingly urgent. Among the most recently undertaken approaches, generation of CAR T cells directly in vivo, although still technically challenging, might be considered a possible innovative alternative to simplify and standardize the manufacturing process, in order to convert CAR-T based therapy into a universally applicable “off-the-shelf” therapeutic product [78]. Moreover, it is reasonable to think that the potential functional exhaustion of current CAR-T cells, due to the several rounds of in vitro expansion and activation prior to their in vivo reintroduction [79, 80], could be overcome, since CAR-T cells reprogrammed directly in situ are unmanipulated and expanded through more gradual kinetics.

As shown in Table 1, in the recent years several groups have reported the successful in vivo generation of CAR T cells in mouse models. To date, systemically injected viral vectors, such as lentiviruses (LV) and Adeno-Associated-Viruses (AAV) and polymer- or lipid-based nanocarriers loaded with CAR-encoding DNA or mRNA have been tested for in vivo generation of CAR-T cells [81–84].

**Table 1:**
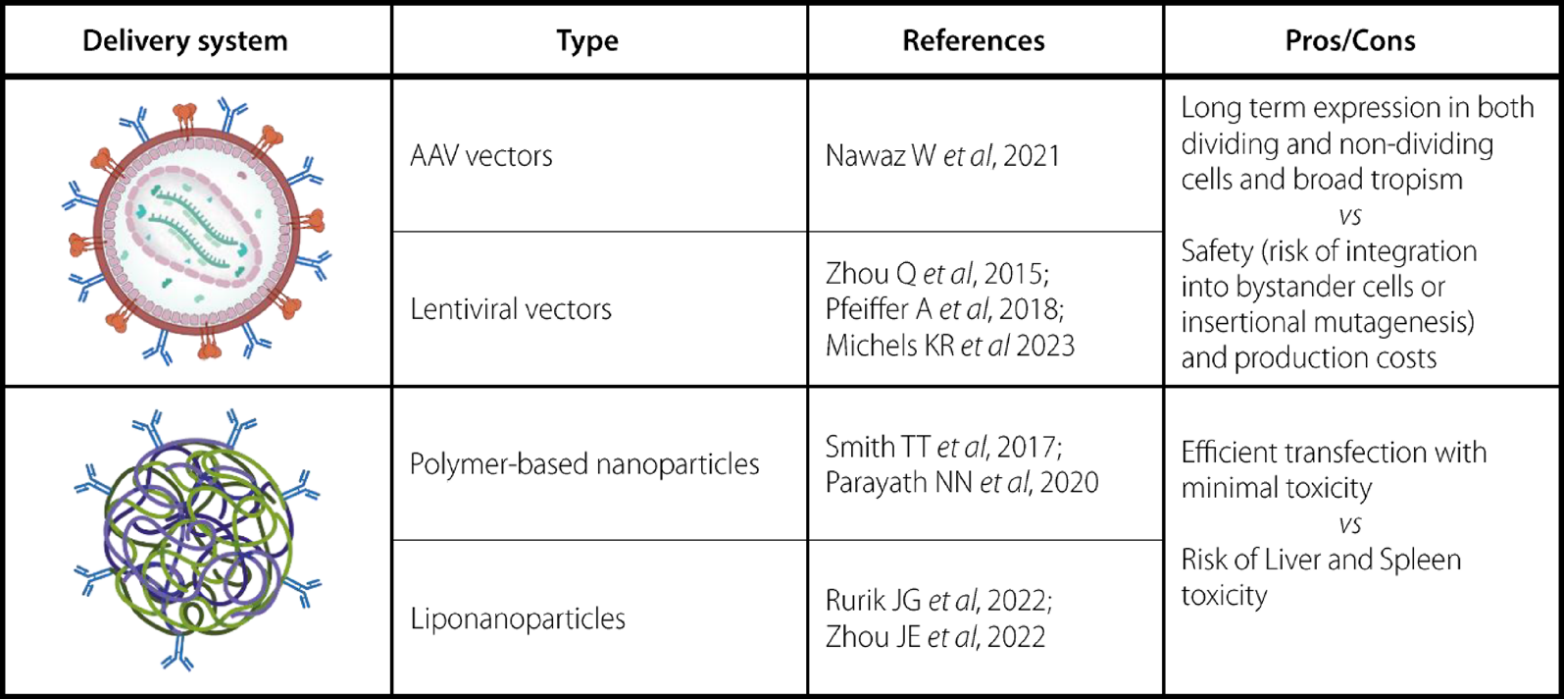
In vivo CAR-T generation systems: type of delivery system, main studies citations and their advantages/disadvantages

## Viral-based delivery systems for in vivo CAR-T generation

Regarding in vivo CAR-T cell therapy, two main classes of viral vectors are being evaluated: AAV vectors and replication-defective lentivirus or retrovirus.

Adeno-associated viruses (AAV) are among the most suitable tools for in vivo gene delivery since long-term expression can be achieved through episomal expression, without integration into the host genome, in both dividing and non-dividing cells and are considered as one of the safest vectors for gene therapy [85].

In the only study regarding the use of AAV vectors for in vivo manufacturing of CAR-T cells, Nawaz et al. observed tumour regression as a result of in vivo reprogramming of immune cells by administrating an AAV carrying a CAR gene [84]*.*

Lentiviral vectors are one of the most common platforms for gene therapy applications, mainly because of the advantages derived by their ability to stably integrate relatively large DNA sequences into host cell genome, their broad tropism and their high transfection efficacy in both dividing and non-dividing cells. Many of the currently clinically approved CAR-T products rely on the use of lentiviral vectors to engineer CAR-T cells ex vivo. Recently, to restrict the tropism of systemically injected CAR-encoding lentiviral vectors to circulating T cells, target ligands such as antibodies, single-chain variable fragment (scFV) or nanobodies directed against T cell surface molecules have been investigated [86, 87]. Pfeiffer et al. demonstrated direct in vivo production of CD19-CAR T cells following intraperitoneal administration of a CD8-scFV fused Nipah virus pseudotyped lentivirus in NSG mice transplanted with human CD34 + cells [88]. Interestingly, Michels et al. developed a lentiviral vector-based platform for in vivo engineering of T cells (VivoVec) with the aim to gain successful CAR T-cell expansion and persistence in vivo, normally ensured by preconditioning with lymphodepleting regimens when ex vivo manufactured CAR-T cells are reinfused into patients [89].

While in vivo CAR-T cell engineering with viral vectors offers several advantages, careful consideration of safety and efficacy is essential to realize its full potential in clinical applications. Indeed, lentiviruses ability to lead to stable integration and expression of the CAR gene not only generates a long-lived cell product but also carries safety risks such as insertional mutagenesis, which can result in dysregulated gene expression in somatic cells and could possibly lead to secondary malignancies. Integration into bystander cells, like germ cells or inhibitory immune cells is also a risk, since it could lead to germline transformation or alter the regulation of the immune response. Moreover, the production of large amounts of GMP-grade lentiviral vectors suitable for human application is a complex and costly process, requiring numerous rigorous tests of the final product to establish purity, potency, and safety, thus increasing production time and the final cost of the therapeutic product. [90, 91].

## Nanoparticle-based delivery systems for in vivo CAR-T generation

Because of the earlier discussed safety concerns, safer and less expensive gene delivery methods for T cell engineering have been investigated, such as electroporation and nanosystems [92]. Currently, electroporation has been employed as physical transfection technique for production of CAR-T cells with mRNA and DNA transposons [93, 94].

Among the non-viral gene delivery strategies, the use of nanosystems to engineer T cells represents an economic and safer gene delivery alternative that can address the challenges of viral- and electrical mechanical-based gene delivery strategies [95]. Moreover, the easy synthesis process and the possibility to storage them in stable forms (e.g. freeze dried) allow mass-production of “ready to use” large amounts of nanosystems for gene delivery. The most frequently incorporated nucleic acid is RNA because of its advantages over DNA. Indeed, RNA is easily synthesized in a cell-free manner and the production process is highly scalable and standardized, thus minimizing the variability of the product and allowing a large-scale manufacturing. Nanosystems can be composed of different biomaterials and the most widely used for CAR T cell engineering are cationic polymers and cationic lipids.

Cationic polymers-based nanosystems used for T cell engineering normally contain Polyethylenimine (PEI), polyethylene glycol (PEG), poly (2-dimethylaminoethyl methacrylate) (PDMAEMA), poly (β-amino esters) (PβAE), polyurethane (PU) and poly-L-histidine (PHSTD) [96, 97, 99]. Moffett HF et al. demonstrated that the use of PBAE polymers loaded with mRNA could efficiently transfect primary T lymphocytes in vitro, with minimal toxicities [100]. By using CAR-encoding plasmid DNA (pDNA) encapsulated in self-assembling nanoparticles (SNPs) composed of PEG, PEI and polyamidoamine (PAMAM), Yu Q et al. demonstrated efficient CAR gene delivery in Jurkat cells with tenfold transfection efficacy compared to Lipofectamine 2000 transfection reagent [101]. Beyond polymer-based nanoparticles, cationic liposomes and lipid nanoparticles (LNPs) represent lipid-based nanosystems commonly used for in vitro T cell engineering.

Liposomes are closed vesicles with an aqueous core surrounded by one or more lipid bilayers of phospholipids, and based on their charge, they can be divided in cationic, anionic or neutral, but cationic ones are the most commonly used for non-viral gene delivery because of their ability to establish electrostatic interactions with the negatively charged nucleic acids [92, 102, 103].

LNPs are composed by a lipid layer containing cationic ionizable lipids, helper lipids, (PEG)ylated lipids and cholesterol, surrounding and nucleic acids [104, 105]. Ionizable LNPs-mediated CAR expression efficacy, and their ability to kill tumour cells in vitro, were found to be similar to those observed in T lymphocytes transfected by electroporation. However, ionizable LNPs-based transfection was accompanied by significative lower toxicity, in terms of T cell viability analysed after transfection [106, 107].

When administering directly in vivo, the risk of liver and spleen toxicity must be considered. Indeed, it has been demonstrated that intravenously injected mRNA-loaded LNPs are endocytosed by various types of cells, mainly hepatocytes [108]. Nonetheless, nanosytems can be easily tailor-made designed to achieve their address to specific target cells, for example by adding target-specific ligands on nanoparticles surface, that selectively bind to T cells and induce receptor-mediated endocytosis [92].

The firsts attempt to achieve in vivo nucleic acid delivery to T cells was carried out by Smith TT [81], by using plasmid DNA encoding leukemic-specific CAR encapsulated in cationic poly (β-amino-ester)-based nanocarriers. In a follow-up study, the same group used PBAE polymer-based nanoparticles loaded with in vitro transcribed (IVT) mRNA for in vivo transient expression of CAR in circulating T lymphocytes. Repeated intravenous administrations of these nanoparticles were able to produce in situ CAR-T cells that shown antitumor responses in xenograft murine models of leukemia, prostate cancer and HBV-induced hepatocellular carcinoma, with efficacies similar to those of adoptive methods [82]. Zhou JE et al. designed LNPs encapsulating pDNA containing human interleukin-6 (IL-6) short hairpin RNA (shRNA) and the CD19-targeting CAR gene [109]. In vivo produced CAR-T cells shown antitumor response against leukemia tumor cells, in xenograft acute lymphoblastic leukemia model, comparable to conventional CAR-T therapy.

In vivo produced CAR-T cells by means of injectable nanosystems loaded with CAR-encoding nucleic acids has been recently investigated also beyond cancer diseases, such as for cardiac fibrosis. Aghajanian H et al. demonstrated that adoptive transfer of T cells expressing a CAR against an endogenous cardiac fibrosis target, fibroblast activation protein (FAP), resulted in reduction of cardiac fibrosis and restoration of myocardium function, in a mouse model of angiotensin II/phenylephrine (AngIi/PE)-induced cardiac injury and fibrosis [110]. Interestingly, Rurik JG et al. used CD5-targeted LNPs encapsulated with FAP-specific CAR-encoding mRNA to engineer circulating T cells and tested the feasibility to mitigate cardiac fibrosis [111]. FAP CAR-T cells were found to accumulate closely to activated fibroblast in injured myocardium and improvements in cardiac function were found 14 days after a single systemic LNPs injection, as measured by echocardiography and histological analyses.

## Possible drawbacks of in vivo T cell engineering

One of the major hurdles of in vivo T cell engineering is to ensure a targeted delivery of CAR transgenes uniquely to T cells, considering that any transduction of immune cells with inhibitory profile (such as regulatory T cells) could compromise the desired antitumor efficacy. More importantly, it is crucial that the CAR-encoding transgene is not delivered into malignant cells: CAR expressed on tumour cell surface could bind to the target epitope in *cis*, thus masking it from detection by CAR-T cells. In a paediatric B- ALL patient treated with ex vivo manufactured anti-CD19 CAR-T cells, transduction of a single leukemic B cell resulted in resistance to antitumor therapy [112]. Several studies have demonstrated that using lentiviruses or nanocarriers expressing ligands such as mAb, scFv or Fab fragment for in vivo T cell engineering significantly increases the delivery of the CAR encoding transgene into T cells, compared to delivery platforms lacking this T cell targeting system [89, 109, 111].

Besides selective gene delivery into T cells, CAR expression persistence overtime in in vivo engineered T cells is another issue to be addressed. Given lentiviral vector ability to stably integrate into the genome of the transduced cell, when employed to generate CAR-T cells in vivo, one single administration of lentiviral vector might ensure highgene transduction efficiency and prolonged CAR expression on engineered T cells [83, 88, 89]. On the contrary, the necessity for repeated administrations of nanocarriers provides the advantage of promptly fine-tune the antitumor potency of CAR-T cells to minimize the potential insurance of systemic toxicities [81, 82].

As previously described, the potential occurrence of systemic toxicities resulting from preconditioning is completely avoided with in vivo CAR-T cells technology. However, other factors, such as the affinity of the antigen-binding domain and the type of costimulatory molecules chosen for CAR design, might lead to excessive activation of in vivo generated CAR-T cells and to the development of systemic toxicities [113]. For example, in ex vivo administered CAR-T cells, it has been demonstrated that CARs with CD28 as costimulatory domain are associated with a more rapid onset of CAR-T cell activity and earlier development of systemic toxicities, compared to CARs with a 4–1BB costimulatory domain [114]. Moreover, in order to enhance CAR-T cells safety, one powerful approach is the introduction of “killing switches”, obtained by adding drug-dependent genes, known as “suicide genes”, such as inducible caspase 9 (iCas9) and Herpes Simplex Virus-1 Thymidine Kinase (HSV- TK), able to trigger apoptosis of CAR-T cell upon administration of the inducing drug [16]. Alternatively, CAR-T cell design can be modified to express elimination markers, such as CD20 or truncated Epidermal Growth Factor Receptor (tEGFR), on their surface. When systemic toxicities occur, administration of mAbs against CD20 (rituximab) or EGFR (cetuximab) can deplete CAR-T cells by ADCC or complement- dependent cytotoxicity (CDC) [115, 116]. The major limitation associated with these inducible death systems is the irreversible loss of CAR-T cells and, in turn, the necessity of a novel manufacturing process whenever the patient still requires further injections of CAR-T cells. To address this issue, scientists have recently developed small-molecule drugs able to reversibly regulate activation or inhibition of CAR-T cells in vivo [117]. Alternatively, the small molecule dasatinib can inhibit the phosphorylation of CD3ζ and ZAP70 kinase, thus blocking CAR signalling in a different manner [118]. Thus, further studies aiming to improve the efficacy and the specificity of the different engineering platforms able to produce CAR-T cells in vivo are warranted.

## Beyond CAR-T cells: in vivo generation of CAR-NK and CAR-M cells

**Fig.3:**
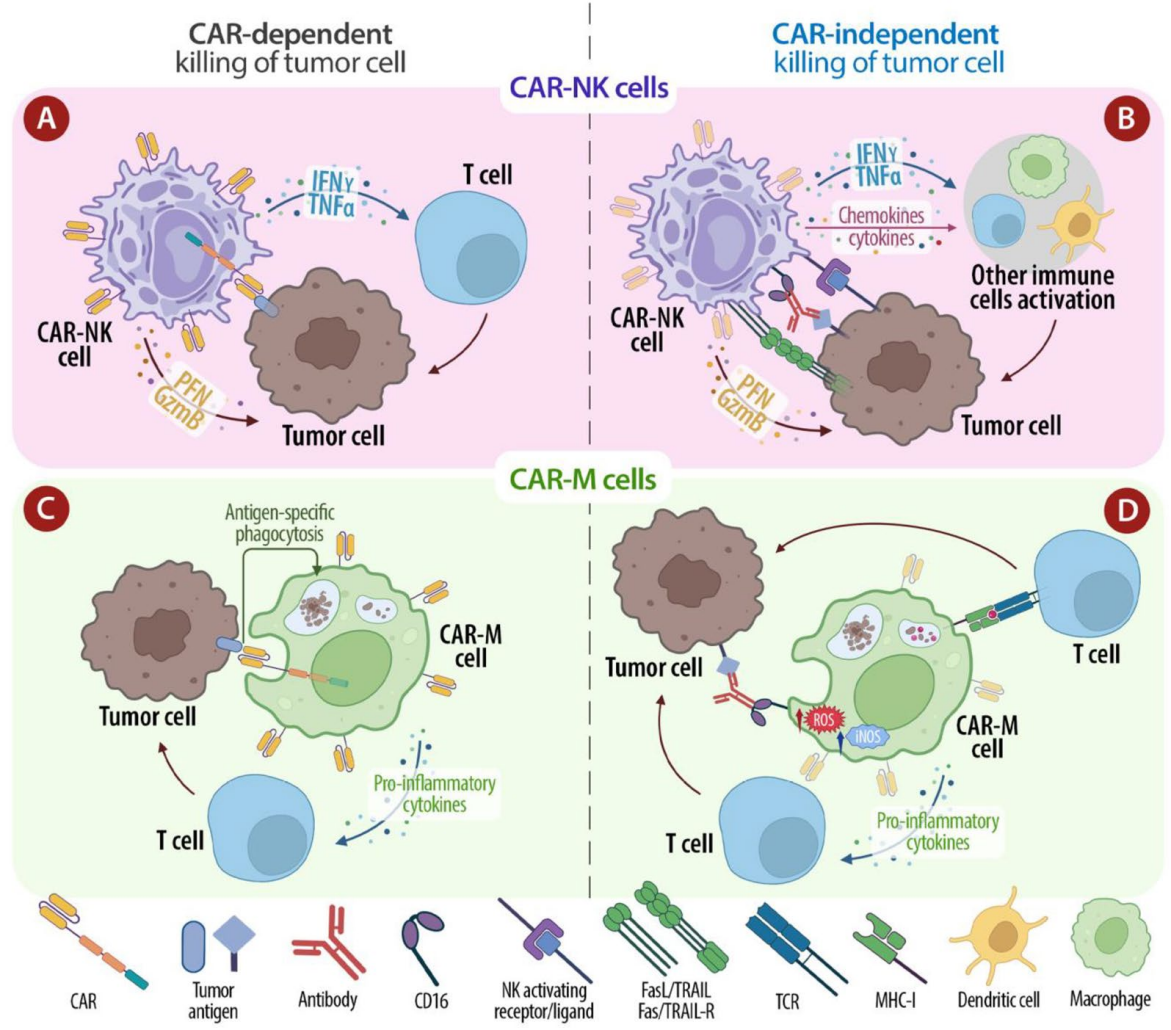
Comparison of CAR-NK and CAR-M cell mechanism of action. **A** CAR-dependent tumor killing pathway of CAR-NK cells involves the binding of a specific tumor antigen with CAR, and the following secretion of perforin and granzyme B, to kill tumor cells, and pro-inflammatory cytokines (TNF-α and INF-γ), to promote CAR-NKs activation and stimulate antitumor response of other T-cells. **B** CAR-NK cells can mediate a direct killing of tumor cells through the following CAR-independent mechanisms: signaling of activating receptors expressed on NK cells surface, that lead to secretion of TNF-α, INF-γ, perforin and granzyme B; induction of cell apoptosis through FasL/Fas and TRAIL/TRAIL-R pathways; triggering of ADCC via the CD16 Fc receptor; secretion of chemokines and cytokines that recruit and activate other immune cells. **C** CAR-dependent tumor killing pathway of CAR-M cells involves the binding of a specific tumor antigen with CAR and the subsequent antigen-specific tumor phagocytosis and release of pro-inflammatory cytokines, which stimulate antitumor response of other T cells. **D** CAR-M cells can mediate a direct killing of tumor cells through the following CAR-independent mechanisms: direct phagocytosis of tumour cells and subsequent presentation of processed tumor antigens to T cells trough MHC-I molecules; secretion of pro-inflammatory cytokines; triggering of ADCC via the CD16 Fc receptor; expression of “killing molecules”, such as ROS and iNOS, which mediate cytotoxic effects on tumor cells

In order to overcome some of the barriers associated with CAR-T cell-based therapy, scientists have investigated the feasibility and the therapeutic effects of other immune cells, such as macrophages and Natural Killer cells (NK), engineered with CARs (Fig. 3).

CAR-M cells are capable of infiltrating solid tumours and withstanding the immunosuppressive tumour microenvironment, potentially reversing it to a more pro-inflammatory state. CAR-NK cells offer the advantage of a lower risk of GvHD and have shown promising antitumor activity with reduced systemic toxicities. These approaches can expand the applicability of CAR therapies to a broader range of cancers and improve patient outcomes.

Regarding the CAR design, most CARs used for NK cell engineering have the same structure as used in CAR-T cells, plus NK-specific intracellular signalling domains, such as NKG2D and DNAX-activation protein (DAP) 10 or DAP12, to improve cytotoxicity [119]. Moreover, it is known that the short lifespan of in vivo transferred NK cells, that might limit their clinical efficacy, can be improved by the presence of cytokines such as IL-2 or IL-15. Therefore, CAR-NK cells persistence and proliferation can be further enhanced by engineering NK cells with fourth generation CARs that autonomously produce IL-2 or IL-15 [120, 121]. Moreover, it was observed in both patients and mouse models that NK cells suppress GVHD by inhibiting T cell activation via their cytotoxic ability and almost all clinical studies on NK cells reported no evidence of GvHD [122]. Therefore, in contrast to allogeneic CAR-T cells which requires a prior step of TCR-gene editing to eliminate the risk of GvHD, thus increasing manufacturing complexity and cost, allogeneic haploidentical CAR-NK cells do not require such gene editing step and can be employed as a “of-the-shelf” ready-to-use product, that can be mass-produced and infused to patients at any time [123]. Beyond the reduced risk of GvHD, another relevant feature that makes CAR-NK cells a safer alternative to CAR-T cells is the lower risk of systemic toxicities, such as CRS and ICANS, frequently occurring in CAR-T cell-based therapy. In addition, the limited in vivo lifetime of the CAR-NK cells is another feature that contributes to reducing the risk of CRS and ICANS. To date, results from several clinical trials demonstrated the safety and efficacy of CAR-NK cell-based therapy and preclinical studies reported CAR-NK cell-mediated antitumor responses markedly higher than those observed with CAR-T cells [124]. To date, the direct in vivo engineering of NK cells with CAR-encoding nucleic acids has been only attempted by Andorko JI and colleagues, who designed a lentiviral vector encoding an anti-CD20 CAR pseudotyped with a novel binder, to provide targeted transduction of CD7 + NK cells following intravenous delivery [125].

An alternative recent approach, especially against solid tumours, is the development of CAR macrophages (CAR-M). In addition to their enhanced ability to infiltrate tumour site, CAR-M cells are capable of withstanding the hostile and immunosuppressive milieu they meet once entered into TME and do not develop an exhausted phenotype, in contrast to CAR-T and CAR-NK cells, whose antitumor function is suppressed by the inhibitory action of both cellular and matric components of TME [126]. In this regard, Klichinsky M et al. demonstrated that HER2-targeting CAR-M cells secreted proinflammatory factors that sustained M2 macrophage polarization toward the M1 phenotype, thus reversing immunosuppressive/protumoral TME toward a proinflammatory/antitumoral state [127]. Once infiltrated into the TME, CAR-M cells, similarly to CAR-NK cells, can exert their cytotoxic function against cancer cells through CAR-dependent and independent pathways, since macrophage ability to directly phagocytize tumour cells and cross-present tumour-derived antigens to T cells, thus increasing specific antitumor T cell response [128]. Similar to CAR-NK cells, CAR-M cells have a low risk of GVHD due to rapid extravasation from blood vessels and in-vivo limited expansion capacity [129]. Moreover, despite the high cytotoxic effects of CAR-M cells, several studies demonstrated lower risk to develop serious side effects, such as CRS and ICANS, but only weak reactions like a low-grade fever, abdominal discomfort, cutaneous toxicity, and body weight loss [130–132].

Alternatively, Treg cells can be engineered with CARs to treat autoimmune diseases. Differently from CAR-T cells, which act through cytolysis, CAR-Treg cells act by suppressing immune responses. Indeed, given to their immunosuppressive functions, adoptive Treg-based immunotherapy has demonstrated promising results in treating several autoimmune diseases or transplant rejection in preclinical models [133–135] and clinical trials. Since the expansion of antigen-specific T cells from the natural Treg pool is technically challenging, a more feasible way is to ex vivo engineer Tregs with recombinant TCRs or CARs. As per CAR-T cells, the main advantage of CAR-Tregs is the complete independence from the MHC presentation by APCs. In addition, it has been demonstrated that CAR-Tregs are less dependent on IL-2 than TCR-Tregs [136]. Currently, ongoing clinical trials cells are testing autologous CAR-Tregs targeting HLA-A2 to prevent organ rejection after liver and kidney transplantation. The efficacy of CAR-Tregs against auto-antigen has been investigated also in several autoimmune diseases. For example, Fransson et al. demonstrated that CAR-Tregs targeting myelin oligodendrocyte glycoprotein (MOG) reduced disease symptoms and decreased proinflammatory cytokine in an autoimmune encephalomyelitis (EAE) murine model [137]. CAR-Tregs against carcinoembryonic antigen (CEA), that has been shown to be overexpressed in ulcerative colitis (UC), showed effectiveness in suppressing the manifestations of colitis in mouse models [133]. Raffin et al. generated CAR-Tregs directed against citrullinated vimentin (CV), which is present abundantly in the extracellular matrix of inflamed joints in patients affected by Rheumatoid arthritis (RA) [138] and a phase I clinical trial is currently investigating the efficacy of autologous CAR-Tregs for treating RA. Additional preclinical studies are assessing the therapeutic potential of CAR-Tregs in other autoimmune diseases, such as vitiligo, asthma and T1D [139–141].

To the best of our knowledge, direct in vivo engineering of Treg cells to produce CAR-Tregs has not been jet investigated. However, in addition to the advantages described for in vivo CAR-T cells, in vivo engineering of CAR-T regs may also solve a safety issue related to the transfer of ex vivo engineered CAR-Tregs into patients, known as “T cell plasticity” [142]. T cell plasticity refers to the ability of T cells to adapt and potentially shift their phenotype and function in response to environmental triggers, such as cytokines or signaling molecules. In the context of Tregs, T cell plasticity may lead to the loss of suppressive functions and covert Treg cells into effector T cells, exacerbating inflammation rather than suppressing it. It has been demonstrated that Tregs easily lose suppressive activity into the artificial environment present during ex vivo expansion [143] so it would be worth to consider direct in vivo production of CAR-Treg cells as a possible strategy to overcome the risk of phenotype shifting, toward pro-inflammatory T cells, of ex vivo expanded CAR-Tregs.

## Conclusions

Despite the impressive clinical outcomes of using CAR-T cells for haematological malignancies, engineering circulating T cells directly in vivo offers a promising alternative. This method can overcome the prolonged time, complexity, and high costs associated with the ex vivo manufacturing of CAR-T cells. Also, beyond the reduction of manufacturing costs and complexity, in vivo CAR-T cell production could allow to overcome also some of the drawbacks associated with patient preconditioning, such the risk of opportunistic infections and systemic toxicities occurrence.

However, some limits associated with the use of systemically administrated lentiviral vectors and nanocarriers must be considered. The main advantage of the use of viral vectors as injectable drug for in vivo reprogramming of circulating T cells, which makes them the gold standard for ex vivo CAR-T cell production too, is their ability to integrate large DNA inserts into both dividing and non-dividing cells, thus promoting stable and long-term surface expression of CARs in transduced cells. Nevertheless, concerns arise form the risk of uncontrollable vector-based insertional mutagenesis and innate immune response against injected viral particles. These limitations can be overcome by systemic administration of non-integrating delivery systems, such as nanocarriers loaded with CAR-encoding DNA or RNA.

Regardless the employed platform, in vivo CAR-T cell manufacturing needs high precision T cell targeting, to avoid modification of other cell types. Additionally, i*n vivo* engineering of other immune cells, such as NK cells and macrophages with CAR-encoding transgene, has shown therapeutic potential. However, in-depth study of strategies to gain specific transgene integration without off-target delivery, and the investigation of consequences deriving from CAR protein expression in non-T cells as well, are definitely warranted.

In vivo CAR-T cell manufacturing also opens the door to broadening the therapeutic applications of CAR technology. For example, the development of CAR-NK and CAR-M cells could offer new strategies for targeting solid tumours and other diseases beyond haematological malignancies. These cells may provide advantages in terms of reduced risk of graft-versus-host disease (GvHD) and potentially lower systemic toxicities.

The integration of in vivo CAR-T cell generation with advanced delivery technologies, such as CRISPR/Cas9 for precise gene editing and novel targeting ligands for enhanced specificity, could further enhance the efficacy and safety of CAR therapies. Such advancements could lead to more precise and controlled modifications, improving therapeutic outcomes.

As the field advances, it is crucial to address regulatory and ethical considerations surrounding in vivo gene editing and CAR-T cell therapies. Ensuring patient safety, informed consent, and equitable access to these innovative treatments will be essential for their successful implementation in clinical practice.

In conclusion, while numerous challenges persist, the encouraging results derived from various preclinical studies hold the promise to concretely consider in vivo CAR-T cell manufacturing as a powerful innovative alternative that, by circumventing ex vivo cell manufacturing, will substantially lower expenses, enhancing accessibility to CAR-T cell therapy for patients requiring this treatment. Nonetheless, comprehensive pharmacokinetic studies and safety validations need to be carried out and must precede the potential application of in vivo CAR-T cell manufacturing platforms in clinical trials. Additionally, expanding the therapeutic applications, integrating advanced technologies, addressing resistance mechanisms, and considering regulatory and ethical implications will be key to the future success of this field.

## Data Availability

Not applicable.
